# Botanical Nanofiber Wound Dressing Loaded with *Psidium guajava* Leaf Extract: Preparation, Characterization, and In Vivo Evaluation

**DOI:** 10.3390/pharmaceutics18010031

**Published:** 2025-12-25

**Authors:** Menna M. Abdellatif, Hesham A. Eliwa, Mohamed Aly Abd El Aziz El Degwy, Samah Shabana, Rafik M. Nassif, Hamada Sadki Mohamed, Rehab Abdelmonem

**Affiliations:** 1 Department of Industrial Pharmacy, College of Pharmaceutical Sciences and Drug Manufacturing, Misr University for Science and Technology, Giza 12566, Egypt; rehab.abdelmonem@must.edu.eg; 2 Department of Pharmacology and Toxicology, College of Pharmaceutical Sciences and Drug Manufacturing, Misr University for Science and Technology, Giza 12566, Egypt; hesham.helmy@must.edu.eg; 3 HiPharm Co., El-Obour City 11828, Egypt; degwys78@hotmail.com; 4 Department of Pharmacognosy, College of Pharmaceutical Sciences and Drug Manufacturing, Misr University for Science and Technology, Giza 12566, Egypt; samah.shabana@must.edu.eg (S.S.); rafik.farajallah@must.edu.eg (R.M.N.); hamada.sadki@must.edu.eg (H.S.M.)

**Keywords:** *Psidium guajava*, freeze drying, botanical nanofiber, wound healing

## Abstract

**Background/Objectives**: This study aimed to develop botanical nanofibers loaded with *Psidium guajava* leaf extract to heal wounds effectively. **Methods**: A 2^3^ factorial design was conducted to study the impact of freeze-drying parameters—freezing time, vacuum, and lyophilization time—on the total phenolic and flavonoid content in the lyophilized extract. Then, a polyurethane-based nanofiber dressing loaded with *Psidium guajava* leaf extract was fabricated using a one-step electrospinning technique. The nanofiber was evaluated considering total polyphenol and flavonoid content, surface roughness, and morphological assessment by scanning electron microscopy. Finally, the nanofiber was evaluated using in vivo wound-healing studies, histopathological analyses, and assessments of tissue levels of tumor necrosis factor-alpha, interleukin-6, matrix metalloproteinase, and growth factors. **Results**: The optimal conditions for freeze-drying the aqueous extract of *Psidium guajava* leaves were a freezing time of 24 h, a vacuum adjusted to 0.02 bar, and a lyophilization time of 48 h. The total polyphenol and flavonoid content within the nanofiber was 96 ± 1.2% and 91.83 ± 2.4%, respectively. Incorporating lyophilized extract in the nanofiber led to a decreased roughness average and root mean square roughness of the nanofiber. The nanofiber was continuous and had a smooth, uniform surface. The in vivo wound-healing assay showed superior wound-healing compared to the commercial Panthenol cream. These results were confirmed with histopathological studies. **Conclusions**: The extraction technique and lyophilization parameters significantly affect the bioactive content of *Psidium guajava* leaf extract. The botanical-loaded nanofiber showed greater wound-healing potential than a commercial cream, confirming its potential in regenerative medicine and wound repair applications.

## 1. Introduction

Wound healing is a highly regulated, multifaceted biological process involving successive yet overlapping phases of inflammation, reepithelialization, and tissue remodeling [1]. Untreated wounds are particularly susceptible to bacterial colonization, which can significantly disrupt the normal progression of tissue repair [2]. According to the World Health Organization, wound infection causes many deaths yearly. This high mortality rate is attributed to poor wound-healing and ineffective wound dressings that fail to eradicate bacteria. Moreover, Mission Regional Medical Center released several research studies and statistics indicating that chronic wounds affect approximately 6.7 million individuals worldwide. The global chronic wound care market is anticipated to expand from USD 12.36 billion in 2022 to reach USD 19.52 billion by 2029 [3]. Therefore, developing more complex and sophisticated wound dressings to replace simple gauze can lead to faster healing and reduced side effects [4]. Wound dressings enhance tissue regeneration through dual mechanisms: firstly, by isolating the wound from external sources of contamination, and secondly, by maintaining a suitably moist microenvironment that supports epithelial renewal. Optimal wound dressings are characterized by their air permeability, efficient absorption of excess wound exudate, and robust protection against microbial invasion. Additional desirable properties include biocompatibility, hypoallergenicity, non-sensitizing effects, and thermal insulation [5].

Recently, wound dressings based on electrospun nanofibers have attracted researchers’ attention due to their scaffolding architecture, which replicates skin characteristics, including the ability to facilitate nutrient infiltration, gas exchange, waste excretion, a porous structure with a high specific surface area, and sustained release of bioactive molecules that could effectively inhibit bacterial proliferation and lower infection risk [6]. The replication of skin characteristics promotes the proliferation and migration of fibroblasts and keratinocytes and enhances collagen synthesis [7,8]. Additionally, the electrospinning method produces thin, continuous, and flexible nanostructures, making them suitable candidates for use in drug delivery systems. Several studies investigated the incorporation of antibacterial agents, growth factors, and other bioactive components into electrospun wound dressings to achieve innovative, multilayered dressings [9,10]. Nanofibers can be classified into four categories based on structural differences: uniaxial, core–shell, porous, and bead-like. Uniaxial nanofibers exhibit outstanding wound-healing performance due to their distinctive physical and chemical characteristics, which facilitate cell adhesion and growth, thereby facilitating tissue regeneration [11]. Among the polymers used to fabricate uniaxial nanofibers is polyurethane, a highly versatile, synthetic, and biocompatible polymer that has attracted significant interest for wound-healing applications due to its remarkable physicochemical properties, such as good barrier and oxygen permeability [12,13].

The standard wound-healing therapies include surgical, non-surgical, and pharmacological treatments. Therapeutic medications such as non-steroidal anti-inflammatory drugs, antiplatelets, corticosteroids, anticoagulants, and vasoconstrictors are frequently used. These drugs possess several adverse effects; for instance, vasoconstrictors negatively impact wound healing by hindering microcirculation, resulting in tissue hypoxia. Therefore, recent investigations have increasingly emphasized the development of innovative resources and technologies to address a wide range of acute and chronic wounds with minimal adverse effects, ease of application, enhanced therapeutic efficiency, and cost-effectiveness for patients.

Botanical nanofibers play a crucial role in wound healing by harnessing the natural bioactive compounds of medicinal plants, which confer anti-inflammatory, antioxidant, and antimicrobial effects essential for tissue repair. Among these, medicinal plants have attracted significant attention as valuable sources of bioactive constituents—particularly flavonoids, terpenoids, and phenolic compounds—that show considerable promise in the management of dermatological conditions [14]. Notably, flavonoids, terpenoids, and phenolic compounds are recognized for their antimicrobial, antifibrotic, antioxidant, anti-inflammatory, analgesic, and tissue regeneration properties. Moreover, a natural antibacterial component can be a more compelling replacement for synthetic particles, such as silver nanoparticles (used in wound dressings), due to its non-toxicity and environmental friendliness [15,16].

Among medicinal plants that are now being utilized for wound care and management is *Psidium guajava* (Guava), a plant with a long-standing presence in Ayurvedic medicine [17]. *Psidium guajava* has been widely used to cure fever, diarrhea, diabetes, hypertension, gingivitis, rheumatism, and inflammation. Additionally, it has been demonstrated that it also accelerates wound healing. This is because *Psidium guajava* leaves contain high levels of bioactive molecules, including terpenoids, tannins, steroids, flavonoids, saponins, and alkaloids [18]. Several studies have demonstrated the therapeutic benefits of *Psidium guajava* leaf extract in healing oral mucosa wounds [19] and diabetic and non-diabetic wounds [20,21].

The extraction method can affect the amount of bioactive compounds extracted from herbs, such as *Psidium guajava* leaves. Therefore, optimizing the extraction method is highly beneficial. Gutierrez Montiel et al. (2023) [22] conducted a study comparing the impact of different extraction methods (Soxhlet, maceration, and ultrasound-assisted extraction) on the phytochemical profile of *Psidium guajava*. They found that Soxhlet extraction with methanol at 65 °C yielded the highest polyphenolic content (up to 44 mg/g) and the most potent antioxidant activity. Soxhlet was advantageous due to its efficiency, time economy, and better reducing power compared to maceration and ultrasound-assisted extraction.

Furthermore, each method produced extracts with different polyphenolic profiles, influencing bioactivity [22]. Moreover, Seo et al. (2014) studied the impact of solvent selection on the extraction of phenolic compounds and flavonoids from *Psidium guajava* leaves [23]. Also, several studies have demonstrated that the stability of polyphenols during extraction is influenced by various factors that can lead to their degradation or transformation. Proper extraction conditions are essential to preserve their antioxidant and therapeutic properties [24].

Lyophilization, also known as freeze-drying, is a dehydration technique employed to preserve thermolabile food products and various biological materials [25]. This method relies on sublimation, which effectively reduces structural changes while preserving the integrity of bioactive compounds and flavor profiles in the dried sample. The lyophilization process involves three principal stages: freezing of the product, primary drying by direct sublimation of ice under reduced pressure, and secondary drying, which removes residual unfrozen water via desorption and diffusion. Lyophilization is considered the gold standard for drying herbal extracts because it occurs at low temperatures under vacuum, thereby limiting oxidative degradation and preserving heat-sensitive compounds such as polyphenols, flavonoids, and essential oils. This process helps maintain the antioxidant activity and chemical integrity of the extract better than conventional drying methods, such as oven drying [26,27].

Therefore, this study aimed to optimize the extraction methods for *Psidium guajava* leaves and to investigate the effects of freeze-drying parameters, namely freezing time, vacuum, and lyophilization time. The optimized conditions were then used to increase the yields of phenolic compounds and flavonoids in the herbal extract. The lyophilized extract was incorporated into uniaxial polyurethane nanofibers, which were fully characterized and evaluated in an in vivo study.

## 2. Materials and Methods

*Psidium guajava* leaves were sourced from MEPACO (Bilbeis, Al-Sharqia, Egypt). Polyurethane was purchased from Sigma-Aldrich (Milan, Italy). TNF-α; Abbexa, (Cambridge, UK), Cat. No: abx050220. IL-6; CLOUD-CLONE CORP, (Katy, TX, USA), Cat. No: SEA079Ra. MMPs; MyBiosource, (San Diego, CA, USA), Cat. No: MBS251552396T. VEGF; CLOUD-CLONE CORP, (Katy, TX, USA), Cat. No: SEA143Mu. TGFB; CLOUD-CLONE CORP, (Katy, TX, USA), Cat. No: SEA124Ra. PDGF; Cell Applications, Inc., (San Diego, CA, USA), Cat. No. CL0485. KGF; LifeSpan Bioscience (Seattle, WA, USA), Cat. No: LS-F11460. EGF; MyBiosource, (San Diego, CA, USA), Cat. No: MBS824918.

### 2.1. Extraction of Polyphenolic and Flavonoid Contents of Psidium guajava Leaves via Maceration Using 70% Hydroalcoholic Solvent

*Psidium guajava* leaves were ground to a 350 g sample using a mechanical grinder, soaked in 70% ethanol (1 L) at room temperature for 7 days, and then filtered. The hydroalcoholic extract of *Psidium guajava* leaves was evaporated using a rotary evaporator at 40 °C to obtain a crude hydroalcoholic extract. Next, column chromatography was used to fractionate the crude extract for the quantitative determination of total polyphenolic and flavonoid contents. Therefore, an aliquot of this crude extract (50 g) was subjected to normal-phase column chromatography on silica gel G-60 packed in a glass column (10 cm i.d. × 60 cm). Elution was performed using a stepwise mobile phase of n-hexane, ethyl acetate, and methanol, increasing in polarity to fractionate the extract and enrich phenolic-containing fractions [23]. Successive column fractions were monitored by thin-layer chromatography (TLC) on silica gel 60 F_254_ plates (20 × 20 cm, Merck, Darmstadt, Germany), following the general TLC approach used for phenolic acids [28]. Briefly, 10 µL of each fraction was applied as bands 1 cm above the lower edge, and plates were developed in a saturated chamber using a phenolic-separation solvent system (n-butanol: acetic acid: water, 4:1:5, *v*/*v*/*v*), as reported for phenolic compounds. The solvent front was allowed to migrate approximately 8 cm, after which the plates were removed, air-dried, and examined under a dual-wavelength UV lamp at 253 and 360 nm to visualize quenching and fluorescent spots corresponding to phenolic constituents. The same plates were then sprayed with a freshly prepared FeCl_3_ solution (2% *w*/*v* in water), and phenolic-positive spots were identified by the appearance of characteristic dark-colored bands. Fractions showing phenolic-positive bands with similar TLC profiles were pooled and evaporated under vacuum to yield phenolic-rich residues, which were used exclusively for the quantitative determination of total polyphenolic and total flavonoid contents using gallic acid and rutin as standards, respectively.

### 2.2. Aqueous Extraction of Polyphenolic and Flavonoid Contents of Psidium guajava Leaves

*Psidium guajava* leaves powder (350 g) was placed in 1 L of distilled water, heated to 80 °C for 30 min, filtered, and lyophilized [29].

### 2.3. Determination of Total Phenolic Content

Total phenolic content was quantified using a microplate-adapted Folin–Ciocalteu assay with gallic acid as the calibration standard, as described by Attard et al. (2013) [29], in both the phenolic-rich fractions obtained from the 70% hydroalcoholic extract and in the aqueous lyophilized extract. A gallic acid stock solution (1 mg/mL in methanol) was prepared, and a series of seven working standards (500, 250, 125, 62.5, 31.2, 15.6, and 7.8 µg/mL) was obtained by serial dilution in methanol. Test samples from each phenolic-rich fraction and from the lyophilized aqueous extract were dissolved in distilled water at 1 mg/mL. For the color reaction, 10 µL of each standard or sample solution was pipetted into the wells of a 96-well microplate, followed by 100 µL of Folin–Ciocalteu reagent (diluted 1:10 with distilled water) and 80 µL of sodium carbonate solution (1 M). The plate was gently mixed using an orbital microplate shaker for 30 s and incubated for 20 min at room temperature in the dark. The absorbance was then measured at 630 nm using a FluoStar Omega microplate reader (BMG LABTECH, Ortenberg, Germany). All measurements were performed in six replicates. A calibration curve was constructed by plotting mean absorbance versus gallic acid concentration, with a coefficient of determination (R^2^) typically of 0.9986, and sample phenolic content was calculated from the regression equation and expressed as mg gallic acid equivalents (GAE) per g extract [30].

### 2.4. Determination of Flavonoid Content

Total flavonoid content was determined in both the phenolic-rich fractions obtained from the 70% hydroalcoholic extract and in the aqueous lyophilized extract using an aluminum chloride microplate assay with rutin as the reference standard, as described by Herald et al. (2012) [30]. Test samples from each phenolic-rich fraction and from the lyophilized aqueous extract were dissolved in distilled water at 1 mg/mL. A rutin stock solution (1 mg/mL in methanol) was prepared, and six working standards (1000, 500, 250, 150, 100, and 50 µg/mL) were obtained by serial dilution. Aliquots of 25 µL of standards or samples were pipetted into the wells of a 96-well microplate containing 100 µL distilled water, followed by 10 µL of 50 g/L NaNO_2_ solution, and incubated for 5 min at room temperature. Subsequently, 15 µL of a 100 g/L AlCl_3_ solution was added, and the mixture was incubated for 6 min; then, 50 µL of 1 mol/L NaOH and 50 µL distilled water were added to each well. The plate was shaken briefly, incubated for 10 min at room temperature, and the resulting red chromophore was measured at 510 nm using a microplate reader. All measurements were performed in six replicates. A calibration curve was constructed by plotting mean absorbance versus rutin concentration, with a coefficient of determination (R^2^) typically of 0.9946, and total flavonoid content was calculated from the regression equation and expressed as mg rutin equivalents (RE) per g of phenolic-rich fraction or aqueous extract [31].

### 2.5. Lyophilization of the Aqueous Extract of Psidium guajava Leaves

The aqueous extract was lyophilized using a freeze-drier (Labconco, Kansas City, MO, USA) and then quantitatively assessed for total polyphenolic and flavonoid content using gallic acid and rutin as standards. A 2^3^ two-level factorial design with two center points and two replicates was conducted to study the impact of freeze-drying parameters—namely, vacuum (X_1_), freezing time at −18 °C (X_2_), and lyophilization time (X_3_)—on total phenolic compounds (Y_1_) and flavonoid compounds (Y_2_). The low and high levels of the three factors were coded as −1 and +1, respectively. Eighteen runs were created, and the design model was a factor interaction (3FI). Model adequacy was evaluated using ANOVA, lack-of-fit testing, residual diagnostics, and comparisons of adjusted and predicted R^2^ and adequate precision values to confirm that the 3FI model provided an acceptable fit and predictive capability for optimization.

### 2.6. Selection of the Optimized Lyophilization Conditions

The optimized lyophilization conditions were selected using Design-Expert software, version 13. The desirability tool was set to select the conditions with the maximum total phenolic and flavonoid content.

### 2.7. Antimicrobial Assessment of Psidium guajava Leaf Extract

The agar-well diffusion assay was performed to assess the antimicrobial activity of *Psidium guajava* leaf extract, which was lyophilized under the optimized conditions [32]. The lyophilized extract was reconstituted in distilled water and then tested for antimicrobial activity. A standardized bacterial inoculum of *Staphylococcus aureus* (*S. aureus*) ATCC 25923 was uniformly spread on a Mueller-Hinton agar (HiMedia, Mumbai, India) surface using sterile cotton swabs. Then, wells were made on the agar surface using a sterile metallic cylinder. Next, 100 μL of reconstituted extract (5 mg/mL) was added to the wells, and the plates were incubated at 35 °C for 24 h. At the end of incubation, the diameters of the inhibition zones formed around the wells were measured. Data is expressed as mean ± standard deviation (SD).

### 2.8. Fabrication of Nanofiber Wound Dressing Loaded with Psidium guajava Leaf Extract

The lyophilized extract of *Psidium guajava* leaves obtained under the optimized conditions was loaded into nanofibers using a locally fabricated electrospinning machine. Using a 500-rpm magnetic stirrer (LX653DMS, LabDex, London, UK), 20% polyurethane polymer was blended with 70 parts tetrahydrofuran (THF) overnight. Two grams of the lyophilized extract were dispersed in THF with the polyurethane polymer. The solution was then transferred into 20-milliliter syringes and placed in the electrospinning device holders. A steady voltage of 20 kV, a tip-to-collector distance of 15 cm, a flow rate of 1 mL/h, a temperature of 25 °C, and a relative humidity of 50% were all maintained during the electrospinning process [13].

### 2.9. Characterization of Nanofiber Wound Dressing Loaded with Psidium guajava Leaf Extract

#### 2.9.1. Determination of Loading Capacity Percent

To determine the loading capacity of nanofibers loaded with *Psidium guajava* leaf extract, three mats of 1 cm^2^ were cut, each immersed in 10 mL methanol, sonicated for 15 min, and then filtered and analyzed for polyphenol content at 630 nm and flavonoid content at 510 nm using a microplate reader. Given that 2 g of extract lyophilized under optimized conditions were incorporated into a 200 cm^2^ nanofiber mat, the theoretical polyphenol content per 1 cm^2^ was calculated as 5.21 mg GAE, and the theoretical flavonoid content per 1 cm^2^ as 1.70 mg RE. The loading capacity was calculated using the following equation:

(1) $$Loading\;capacity\;\%=\frac{Actual\;content\;of\;polyphenols\;or\;flavonoids}{Therotical\;content\;of\;polyphenols\;or\;flavonoids}\;\times 100$$

Data is expressed as mean ± SD.

#### 2.9.2. Morphology Characterization

The morphology of the botanical-loaded nanofiber was evaluated by scanning electron microscopy (SEM) analysis. A SEM (JSM-6301 F, JEOL Inc., Peabody, MA, USA) was used to examine gold-coated samples and explore the surface characteristics of the compositions. The accelerating voltage was 20–30 kV [33].

#### 2.9.3. Determination of Surface Roughness Using Atomic Force Microscopy

After SEM analysis, the surfaces of unloaded nanofibers and nanofibers loaded with *Psidium guajava* leaf extract were scanned using Gwyddion 2.31 to assess roughness for loaded and unloaded nanofibers. The resolution of the 3D micrograph module was fixed at 1400 × 960 pixels to facilitate comparison between them. The edges of the photomicrographs have been eliminated to prevent unnecessary limit values. Consequently, the dependence of the roughness parameters on the deviation in composition was anticipated in (nm) utilizing the same software [34].

#### 2.9.4. Determination of Swelling Index

The weight of the nanofiber loaded with extract was measured before and after soaking in a phosphate-buffered saline with a pH of 7.4 for 24 h, and the swelling index was calculated through the following equation [35]:

(2) $$S\%=\frac{Ws-Wd}{Wd}\times 100$$

where: *S*% = swelling index (%), *Ws* = swollen weight (g), and *Wd* = dry weight (g).

Data is expressed as mean ± standard deviation (SD).

#### 2.9.5. Mechanical Properties

Tensile properties, including tensile strength and tensile modulus of the nanofiber loaded with extract, with dimensions of 10  ×  30 mm, were determined using a Multi-Test 2.5-i tensile testing machine (2.5-i, Mecmesin Ltd., Slinfold, UK). The tensile test was performed at a rate of 5 mm/min using a 50 N load cell at 25  ±  1 °C. Finally, the information on tensile properties was collected from the load–extension curve and analyzed. The membrane thickness was measured using a digital micrometer. Data is expressed as mean ± (SD).

#### 2.9.6. BET Surface Area and Porosity Analysis

The nanofibers’ specific surface area and pore size were measured using a Nitrogen Adsorption/Desorption analyzer (BELSORP Max, MicrotracBEL Corp., Osaka, Japan) at 77 K. Before the test, the sample was degassed for 12 h under vacuum [36]. Nitrogen adsorption–desorption isotherms were recorded, where the Brunauer–Emmett–Teller (BET) method was employed to determine the specific surface area from the linear region of the BET plot. At the same time, the pore size and pore volume were calculated using the Barrett-Joyner-Halenda (BJH) method. Additionally, the pore size distribution was estimated utilizing Non-Local Density Functional Theory (NLDFT).

### 2.10. In Vivo Study

#### 2.10.1. In Vivo Wound Healing Assay

Fifty-four Sprague–Dawley male rats without skin damage or diseases were obtained from Misr University for Science and Technology (MUST) animal center, weighing between 150 and 200 g, and underwent dorsal full-thickness excisions measuring 4 × 3 cm^2^ under anesthesia induced by intraperitoneal administration of pentobarbital at a dose of 50 mg/kg [16]. The experimental protocols were approved by the Ethical Committee of the College of Pharmaceutical Sciences and Drug Manufacturing at MUST (IP05). All procedures conformed to the guidelines for the care and use of laboratory animals established by the US National Institutes of Health (publication No. 85–23). The animals were allocated to three groups of 18 rats each using a random number table, as detailed in Table 1. The number of animals was determined depending on the primary outcome variable and the expected treatment effect.

Treatments were applied topically to the wounds once daily for a duration of 10 days, with care taken to avoid wound trauma. Wound surface areas were assessed by radial planimetry on days 5 and 10 post-injury. Throughout the study, rats were housed individually in wire-bottom cages under a 12 h light/dark cycle and provided with standard chow and water ad libitum. The investigators responsible for group allocation and treatment administration were aware of the group assignments; however, those responsible for outcome evaluation and data analysis remained blinded to the group assignments during these stages of the experiment. The percentage of wound closure was calculated using the following formula [16]:

*Wound closure* (%) = (*Wt*_0_ − *Wt_i_*)/*Wt*_0_ × 100 (3)

*Wt*_0_ is the initial wound area at the experiment’s outset, and *Wt_i_* is the measured wound area at a given time interval.

#### 2.10.2. Histopathology

Skin tissue samples at the wound area were dissected out and kept in neutral formalin (10%) on the 5th and 10th days after treatment from three scarified animals from each group (*n* = 3 per time). Tissue samples were then routinely processed by passing through graded alcohols, clearing in xylene, and embedding in paraffin wax. Paraffin tissue blocks were cut into 5 µm sections, dewaxed in xylene, rehydrated, and stained with hematoxylin and eosin (H&E) [37]. A score ranging from 0 to 4 was assigned to each of the following criteria: degree of reepithelialization, granulation tissue and collagen matrix organization, and degree of inflammation and angiogenesis, as described by Bakr et al. (2021) [38]. To evaluate collagen content in the wound area, tissue sections were stained with Masson’s trichrome (MTC), and the stained areas were quantified as area% using CellSens Dimensions (Olympus software, version 1.7). Q-Q plots and the Levene test were used to assess normality and homogeneity of variance, and a one-way ANOVA followed by Tukey’s multiple-comparison post hoc test was used to assess differences among groups. The data were expressed as mean ± standard error of the mean (SEM). Statistical analysis of the outcomes was performed using Prism Instat software, version 8 (GraphPad Software, San Diego, CA, USA).

#### 2.10.3. Detection of Cytokines and Growth Factors

Skin tissue samples at the wound area were dissected out on the 5th and 10th days after treatment from six rats from each group (*n* = 6). Then, tissues were rinsed thoroughly in ice-cold phosphate-buffered saline to remove residual blood and weighed prior to processing. Samples were minced into small pieces and homogenized on ice in fresh lysis buffer at a 1:20 *w*/*v* ratio, corresponding to 1 mL of buffer per 20 mg of tissue. The homogenates were then sonicated using an ultrasonic cell disruptor until clarified. The resulting suspensions were centrifuged at 10,000× *g* for 5 min at 4 °C. Supernatants were collected and either used immediately for assays or aliquoted and stored at –20 °C until further analysis. Tumor necrosis factor-alpha (TNF-α), Interleukin-6 (IL-6), Matrix metalloproteinases (MMPs), Vascular endothelial growth factor (VEGF), Transforming growth factor (TGFB), Platelet-derived growth factor (PDGF), Keratinocyte growth factor (KGF), and Epidermal growth factor (EGF) were measured using ELISA according to the manufacturer’s instructions. The assay procedure was followed as described in the manuals, and the absorbance of the yellow color was measured at 450 nm. The aliquot concentrations were determined from the corresponding standard curve. Q-Q plots and the Levene test were used to assess normality and homogeneity of data. The data were expressed as mean ± SD. A one-way Welch ANOVA followed by Games-Howell post hoc tests in Prism Instat software, version 8, was used to assess cytokine and growth factor levels statistically.

### 2.11. Statistical Analysis of Data

The impact of independent variables (lyophilization conditions) on total phenolic and flavonoid contents was analyzed using Design-Expert software, version 13. A 3FI factorial model was fitted, and analysis of variance (ANOVA) was used to test the significance of main effects and interaction terms at a significance level of *p* < 0.05. For statistical analysis of histopathological scores, a one-way ANOVA followed by Tukey’s multiple-comparison post hoc test was used to assess differences among groups. For statistical analysis of cytokines and growth factors, A one-way Welch ANOVA followed by the Games-Howell post hoc test was used.

## 3. Result and Discussion

The extraction procedure aims to maximize the yield of bioactive chemicals from the material while maintaining both its functional and structural integrity [39]. Numerous variables, including extraction time, temperature, solvent type, and concentration, influence the extraction of plant bioactive compounds. Selecting the appropriate solvent type and optimal conditions for each plant sample is essential for achieving maximum yield of each natural compound [40].

### 3.1. Determination of Polyphenolic and Flavonoid Contents of Psidium guajava Leaves via Maceration Using 70% Hydroalcoholic Solvent

The total polyphenolic content of the 70% hydroalcoholic extract of *Psidium guajava* leaves was quantified as 179.16 ± 2.1 mg GAE/g, while the flavonoid content was measured at 84.23 ± 1.8 mg RE/g. Maceration, a traditional extraction method, operates on the principle of passive diffusion, in which soluble compounds are transferred from plant tissues into the solvent. During this process, the solvent permeates the plant’s cellular structure, dissolving the target phytochemicals, which then migrate along a concentration gradient. Despite its simplicity and minimal technological requirements, maceration is characterized by longer processing times and lower extraction efficiency than modern extraction methods. Nevertheless, it remains widely employed due to its accessibility and straightforward procedure [41].

### 3.2. Determination of Polyphenolic and Flavonoid Contents of Aqueous Extract of Psidium guajava Leaves

The decoction technique, a traditional extraction method, involves heat treatment that breaks down plant cells, allowing the release of bioactive components into the aqueous phase. The polyphenol content ranged from 405.928 ± 2.2 to 520.756 ± 2.8 mg GAE/g, while flavonoids ranged from 119.75 ± 1.1 to 208.25 ± 1.5 mg RE/g, depending on lyophilization parameters. Although previous studies have shown that aqueous–ethanol mixtures can be more efficient than pure water for phenolic extraction across different plant matrices [42,43], the current study highlights the impact of solvent composition and extraction temperature on the bioactive yield of the plant, where the optimal procedure is usually different for different plant matrices and depends on the polyphenol composition [44]. The higher polyphenol and flavonoid content obtained with the hot aqueous extraction at 80 °C can be explained by the combined effect of elevated temperature and high solvent polarity. The elevated extraction temperature might enhance solubility, increase diffusion coefficients, and reduce the solvent’s viscosity and surface tension, thereby facilitating mass transfer and improving extraction efficiency [45,46]. Furthermore, water at high temperature can efficiently extract more hydrophilic phenolic acids and flavonoid glycosides that might be left behind by 70% ethanol [47]. Also, it can facilitate the release of conjugated or bound phenolics, as high temperatures can break bonds between polyphenolic compounds and macromolecules, such as proteins, thereby increasing the concentration of free phenolics in the extract [48]. This may explain the better performance of the hot aqueous extraction compared to the 70% hydroalcoholic extraction in this study. These results agree with Baron et al., who found that water extraction under heating conditions of red grape skin is effective (extraction yields are similar or even better than those with a binary solvent) for some polyphenolic classes, such as hydrophilic procyanidins, phenolic acids, flavonol glucosides, and stilbenoids [49]. Similarly, Lorena et al. reported that a decoction of *Psidium guajava* leaves (hot aqueous extract) yielded notably high total phenolic (89.6 µg GAE/mg) and flavonoid contents (749 µg RE/mg), along with potent antioxidant capacity (EC50 = 7.45 μg/mL) [50]. These findings indicate that hot aqueous extraction is particularly effective for extracting hydrophilic and conjugated phenolics. Furthermore, lyophilization operates at very low temperatures under vacuum, preserving heat-sensitive phenolics and flavonoids while minimizing oxidation and enzymatic degradation that often occur during vacuum drying at elevated temperatures. This leads to higher recovery and retention of intact bioactive compounds [26].

In the current study, a 2^3^ two-level factorial design was conducted to examine the impact of freeze-drying parameters—namely, vacuum (X_1_), freezing time at −18 °C (X_2_), and lyophilization time (X_3_)—on total phenolic compounds (Y_1_) and flavonoid compounds (Y_2_). The constructed factorial models were verified by comparing model-predicted responses with experimentally measured values using diagnostic plots, which showed good agreement. Moreover, the adjusted and predicted R^2^ values differed by less than 0.2, supporting the models’ predictive capability. In addition, adequate precision values greater than 4 indicated a satisfactory signal-to-noise ratio, showing that the models were suitable for reliable optimization of the lyophilization conditions. The runs are presented in Table 2, and the design output is shown in Table 3.

#### 3.2.1. The Impact of Vacuum on the Polyphenols and Flavonoids Content of Lyophilized Aqueous Extract

**Figure 1:**
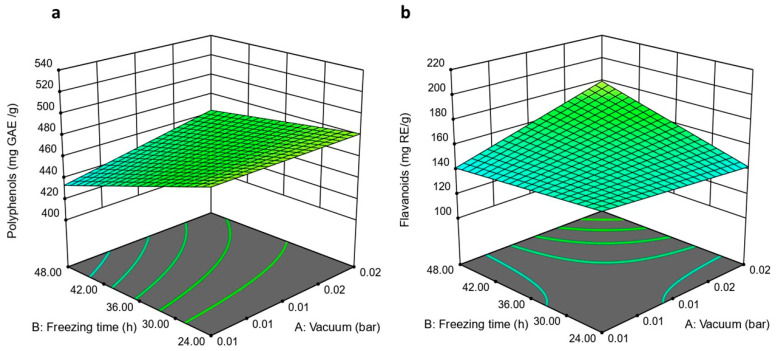
The impact of vacuum and freezing time on (**a**) polyphenol and (**b**) flavonoid content.

The vacuum had a significant impact on polyphenol (*p* = 0.0151) and flavonoid (*p* = 0.0108) content, as shown in Figure 1. Varying the vacuum from 0.01 bar to 0.02 bar resulted in a substantial increase in polyphenol and flavonoid content. Notably, the impact on flavonoid content was most pronounced at prolonged freezing time, with the extract lyophilized at 0.01 bar for 48 and 24 h of freezing and lyophilization, respectively, yielding 405.92 ± 2.2 mg GAE/g polyphenols and 122.59 ± 2.5 mg RE/g flavonoids. At the same time, extracts lyophilized under similar freezing and lyophilization times, but with a varied vacuum of 0.02 bar, showed 451.00 ± 0.2 mg GAE/g polyphenols and 166.13 ± 2.5 mg RE/g flavonoids. Lyophilization at higher pressure increases the sublimation temperature and, consequently, reduces the complex viscosity of the frozen material, leading to accelerated shrinkage, an increase in apparent density, and a decrease in porosity. These physical changes facilitate improved retention and possible release of polyphenols and flavonoids, as documented in food and botanical freeze-drying studies [51]. Moreover, lyophilization at 0.01 bar resulted in sublimation occurring at very low product temperature. However, with a high driving force for mass transfer, this can lead to significant structural stress and greater ice crystal damage [52]. On the contrary, when the pressure is modestly increased to 0.02 bar, the sublimation temperature increases by a few degrees, and the primary drying rate becomes less extreme. The product then follows a slightly higher but more stable temperature profile, leading to a shorter or smoother drying step and reduced structural stress. Hence, a larger fraction of phenolics is retained in the final powder.

#### 3.2.2. The Impact of Freezing Time on the Polyphenols and Flavonoids Content of Lyophilized Aqueous Extract

Freezing time negatively affected the polyphenol content of the lyophilized extract (*p* ≤ 0.0001). The extract lyophilized at 0.02 bar, with freezing and lyophilization times of 48 h, showed 477.08 ± 1.7 mg GAE/g polyphenol. At the same time, extract lyophilized under similar vacuum (0.02 bar) and lyophilization time (48 h), but with a shorter freezing time (24 h), showed a higher polyphenol content (520.75 ± 2.8 mg GAE/g). These results might be due to polyphenol oxidase (PPO) and peroxidase (POD), which enzymatically degrade polyphenols. Korbel et al. studied the activity of PPO and POD in mango. They found that these enzymes remain active at lower temperatures and remain partially active at the end of the drying process. Similarly, studies on other fruits, such as peaches and papayas, have shown that PPO retains some activity at subzero temperatures and contributes to polyphenol degradation during frozen storage or freeze-drying [53]. Moreover, because the extract obtained by the decoction method might still contain cell wall fragments, membranes, proteins, and bound phenolics, prolonged freezing could also promote recrystallization and growth of ice crystals, mechanically disrupting cell and matrix structures and releasing pro-oxidant enzymes that further degrade phenolics before drying starts [54]. These facts could explain the reduction in polyphenol content observed with prolonged freezing time.

On the contrary, freezing time positively affected flavonoid content (*p* = 0.0153). The extract lyophilized at 0.02 bar, with freezing and lyophilization times of 48 h, showed 208.25 ± 1.5 mg RE/g flavonoid. At the same time, extract lyophilized under similar vacuum (0.02 bar) and lyophilization time (48 h), but with a shorter freezing time (24 h), showed lower flavonoid content (169.54 ± 3.6 mg RE/g). This positive effect could arise from enhanced extractability or stability of flavonoids under freezing conditions, as reported in some freeze-drying studies, where flavonoid levels increased due to inactivation of specific enzymatic or non-enzymatic pathways that degrade flavonoids during lyophilization [55]. Moreover, freezing-induced cell disruption and ice crystal damage can further release bound or compartmentalized flavonoids into the extractable phase, thereby increasing their content in the lyophilized extract [56].

#### 3.2.3. The Impact of Lyophilization Time on Polyphenols and Flavonoids Content of Lyophilized Aqueous Extract

**Figure 2:**
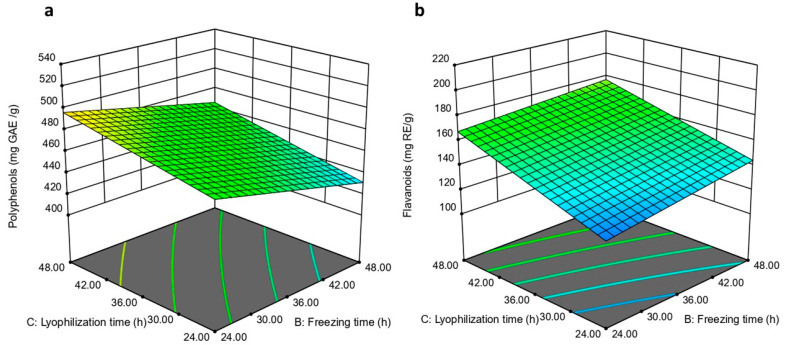
The impact of lyophilization time and freezing time on (**a**) polyphenol and (**b**) flavonoid content.

Extending the lyophilization time increased the polyphenol and flavonoid content of the lyophilized extract (*p* ≤ 0.0001), as shown in Figure 2. Extract lyophilized under conditions of 0.02 bar vacuum, freezing time 48 h, and lyophilization time 48 h showed higher polyphenol (467.16 ± 3.5 mg GAE/g) and flavonoid (176.22 ± 3.0 mgRE/g) compared to extract lyophilized under similar conditions but with shorter lyophilization time (458.28 ± 4.9 mg GAE/g and 169.04 ± 2.1 mgRE/g, respectively). Prolonged lyophilization improves the preservation and stability of phenolic and flavonoid compounds by decreasing residual moisture, thereby reducing enzymatic and non-enzymatic degradation pathways that typically require water. This extended lyophilization time also helps to maintain better the molecular structure of sensitive compounds like polyphenols and flavonoids, which can be degraded or transformed during insufficient or rapid drying. Previous studies have reported increases or better retention of phenolic content and antioxidant capacity with optimized lyophilization durations compared to shorter cycles [26,57,58].

### 3.3. Selection of the Optimized Lyophilization Conditions

The desirability tool was set to select conditions that maximized total polyphenol and flavonoid compounds in the extract, with conditions chosen as 0.02 bar vacuum, 24 h freezing time, and 48 h lyophilization time, resulting in a desirability of 0.770. The extract lyophilized under these conditions showed the highest polyphenol content (520.75 ± 2.8 mg GAE/g) and moderate flavonoid content (169.54 ± 3.6 mg RE/g).

### 3.4. Antimicrobial Assessment of Psidium guajava Leaf Extract

The *Psidium guajava* leaf extract showed an inhibition zone of 31.05 ± 0.99 mm against *S. aureus* ATCC 25923, indicating potent antibacterial activity. The well-documented antibacterial activity of *Psidium guajava* leaf extract against Gram-positive bacteria is attributed to its flavonoid, polyphenol, and tannin content. Flavonoids, such as guaijaverin and quercetin, present in *Psidium guajava* leaves, form complexes with extracellular proteins, dissolve in bacterial cell walls, and disrupt the bacterial life cycle. Tannins, which are polyphenolic substances, inhibit protein synthesis and cause protein denaturation, ultimately impairing bacterial metabolism; tannins can also damage bacteria by solubilizing the lipid components of the cell envelope, leading to leakage of cellular contents and cell death [59].

### 3.5. Characterization of Nanofiber Wound Dressing Loaded with Psidium guajava Leaf Extract

#### 3.5.1. Determination of Loading Capacity Percent

The extract showed a total polyphenol content of 520.765 mg GAE/g extract and a total flavonoid content of 169.54 mg RE/g extract, utilizing optimized lyophilization parameters (Run 8). Given that 2 g of extract were incorporated into a 200 cm^2^ nanofiber mat, the theoretical polyphenol content per 1 cm^2^ was calculated as 5.21 mg GAE, and the theoretical flavonoid content per 1 cm^2^ as 1.70 mg RE. The total polyphenol and flavonoid content within the nanofiber was 96 ± 1.2% and 91.83 ± 2.4%, respectively. The results were within the desired range of 90–110%, suggesting that the extract was uniformly dispersed within the nanofiber matrix.

#### 3.5.2. Morphology

**Figure 3:**
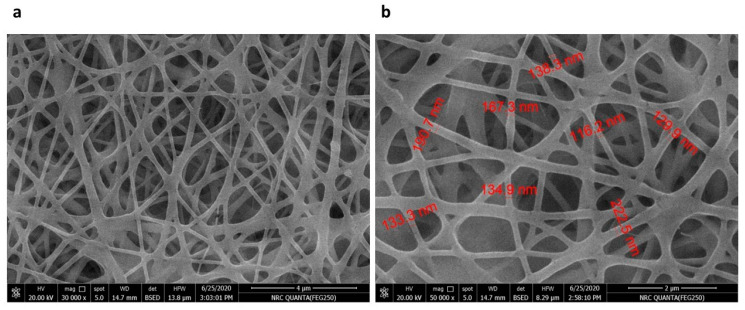
The SEM images of nanofibers loaded with *Psidium guajava* leaf extract: (**a**) surface morphology at 30,000× showing uniform nanofiber formation and smooth texture; (**b**) higher magnification at 50,000× revealing fiber diameters.

SEM images of the nanofiber loaded with *Psidium guajava* leaf extract showed a fibrous morphology comparable to that of the pristine polyurethane nanofibers, indicating that extract incorporation did not compromise fiber formation. The fabricated fibers had smooth, continuous, and homogeneous structures, as shown in Figure 3a. Figure 3b demonstrates the fiber diameters.

#### 3.5.3. Determination of Surface Roughness

**Figure 4:**
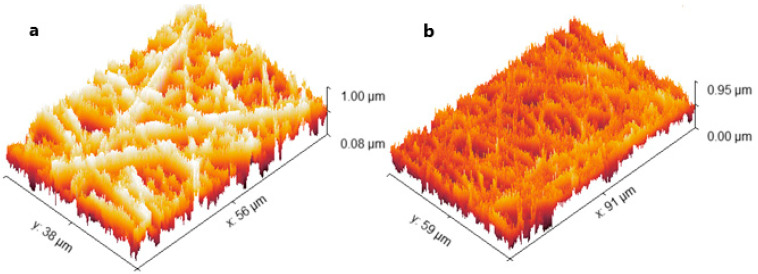
The surface parameters of (**a**) unloaded fiber and (**b**) loaded fiber.

Table 4 and Figure 4 depict the surface parameters before and after loading the extract. Incorporating the extract resulted in a modest decrease in the roughness average (R_a_) value, from 49.37 to 45.36 nm, indicating a slight smoothing or increased uniformity of the fiber surface. This suggests that the active compounds in the extract were well dispersed and embedded within the fiber matrix, thereby reducing surface irregularities. As expected, the root-mean-square roughness (R_q_) followed a similar trend, decreasing concurrently with R_a_.

The values of maximum height of the roughness (R_t_) decreased from 522.49 nm in the unloaded fibers to 494.47 nm after loading, reflecting a reduction in the maximum height difference across the surface profile. Trends in the maximum roughness peak height (R_p_) and the average maximum roughness height (R_tm_) follow the same pattern, with lower values post-loading, indicating a narrower range of surface irregularities. The divergence in the behavior of R_a_ and R_q_ compared to R_t_ and R_p_ highlights the different aspects these parameters quantify: R_a_ and R_q_ represent average surface deviations, while R_t_ and R_p_ measure extreme features such as peaks and valleys.

This divergence suggests a surface with prominent peaks and valleys, which enhance adhesion and are beneficial for cell attachment and growth, particularly in tissue engineering applications. A higher degree of surface roughness, characterized by irregularities like peaks and valleys, can enhance mechanical interlocking and chemical interactions with surrounding tissues or cell membranes. These results align with the existing literature, which indicates that increased surface roughness at the micro- and nanoscale levels encourages cell adhesion and proliferation, making it advantageous for biomedical scaffolds.

#### 3.5.4. Determination of Swelling Index

A promising wound dressing material should be able to absorb excess exudate without causing wound desiccation. Additionally, it must be nonadherent to allow painless removal without damaging newly formed skin. The swelling index of the nanofiber loaded with extract was 279 ± 5.1%, and this value can provide sustained exudate absorption for a considerable period, helping prevent septic shock. Meanwhile, it can also maintain a moist environment required for effective management of burn wounds. The high swelling index of the nanofiber indicates its promising potential as a wound dressing material with both protective and therapeutic benefits, consistent with findings from other studies on polyurethane nanofibers loaded with natural extracts for wound healing [60].

#### 3.5.5. Mechanical Properties

The nanofiber mat loaded with *Psidium guajava* leaf extract exhibited a tensile strength of 1.05 ± 0.1 MPa and an elastic modulus of 2.9 ± 0.04 MPa, reflecting a soft, easily deformable structure. Previous studies have shown that nanofiber mats with tensile strengths between 1 and 3 MPa have been successfully developed and applied for wound healing [61]. Moreover, the elastic modulus is well-suited for wound-dressing applications, as it closely matches that of human skin, which typically ranges from 0.1 to 10 MPa [62]. The elongation at break of the nanofiber was 15.5 ± 1.13%, indicating that the mat has moderate extensibility, allowing some stretching before failure and reasonable flexibility without marked brittleness [63]. Electrospun nanofibers designed for wound dressings have shown elongation at break values from 8% to 91%, with many previous studies considering these ranges suitable for skin wound applications [64,65]. The average thickness of the nanofiber was 0.09 ± 0.002 mm, consistent with previous studies showing that nanofibers up to 0.5 mm correlate with improved porosity and exudate absorption, supporting the suitability of thicker nanofiber mats for wound-healing applications [66]. Overall, these data characterize the nanofiber-loaded *Psidium guajava* as a soft, flexible nonwoven mat associated with increased compliance and reduced stiffness. Such compliance is desirable for wound dressings, as it allows the material to conform closely to the skin and wound bed, enhancing comfort and intimate contact [67].

#### 3.5.6. BET Surface Area and Porosity Analysis

**Figure 5:**
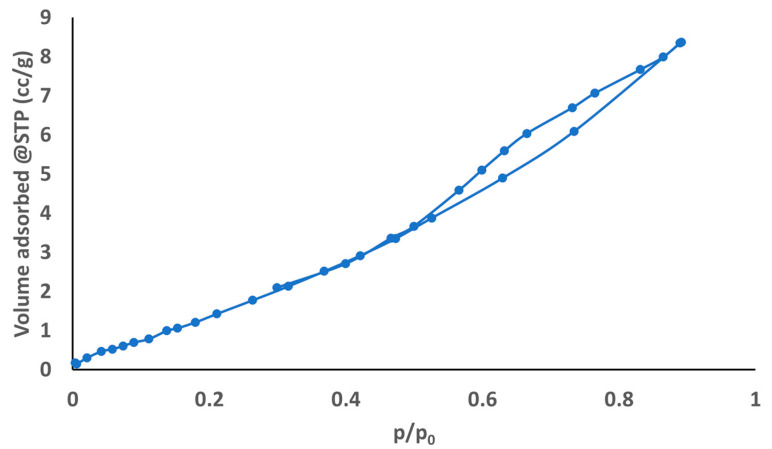
Nitrogen adsorption–desorption isotherm of *Psidium guajava* leaf extract-loaded nanofiber.

Nitrogen adsorption analyses (BET, BJH, NLDFT, and isotherm) show that the *Psidium guajava* leaf extract-loaded nanofiber is a moderately porous mesoporous scaffold with a narrow pore size distribution. The BET surface was 12 m^2^/g, with a total pore volume of about 0.074 cm^3^/g and a mean pore diameter around 24.58 nm, which is confirmed by both BJH and NLDFT pore size distribution analyses. The BJH analysis showed BJH surface area of 13.072 m^2^/g, peak pore diameter of 21.80 nm, and BJH desorption pore volume of 0.7382 cm^3^/g. The pore volume from NLDFT (0.0781 cm^3^/g) is very close to the BJH/BET total pore volume, with a peak pore width of 24.557 nm. The adsorption–desorption isotherm (Figure 5) displays the gradual uptake and capillary condensation characteristic of mesoporous materials, with minimal hysteresis, suggesting relatively open pores. These results suggest that the nanofiber can absorb fluids (exudate) and facilitate the diffusion of *Psidium guajava* leaf extract components, which is appropriate for wound-dressing applications.

### 3.6. In Vivo Study

#### 3.6.1. In Vivo Wound Healing Assay

**Figure 6:**
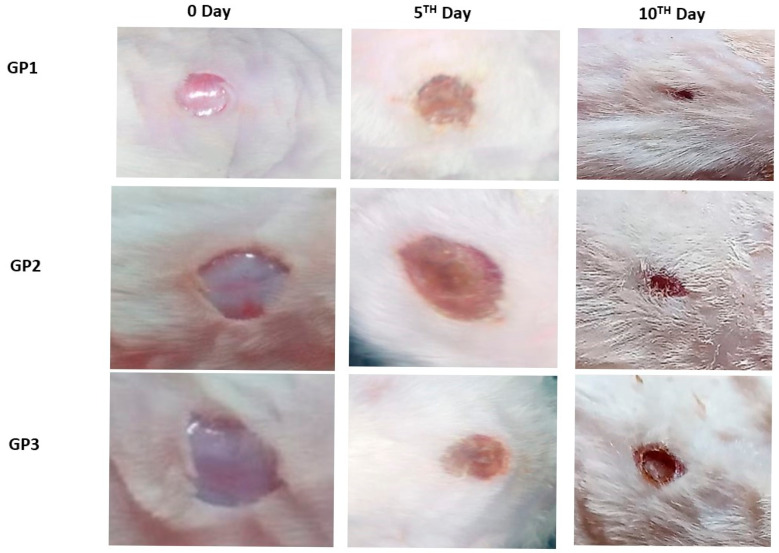
Photographs displaying wound diameters in different groups on the 5th and 10th day after treatment.

The results from in vivo wound-healing showed that the nanofiber loaded with *Psidium guajava* leaf extract (GP1) led to a significant increase in wound closure percentage compared to the group treated with commercial Panthenol cream (GP2) or the control group treated with distilled water (GP3) at all time intervals, as illustrated in Figure 6. *Psidium guajava* leaves contain high levels of bioactive molecules, including terpenoids, tannins, steroids, flavonoids, saponins, and alkaloids, and they have been shown to accelerate wound healing by promoting epithelialization and fibroblast proliferation, reducing oxidative stress, and modulating inflammatory responses. Additionally, *Psidium guajava* leaves constituents exhibit broad-spectrum antibacterial activity against Gram-positive and Gram-negative bacteria, helping prevent wound infections. Our findings agree with those of Fernandes et al., who reported that *Psidium guajava* leaf extract reduced cellular viability and the growth of microorganisms in vitro compared with the control group. At the same time, in vivo, it accelerated wound healing [68]. Furthermore, in various experimental models—ranging from excision wounds in rats to gingival tissue repair—*Psidium guajava* leaf extract has consistently led to earlier scar formation, increased fibroblast proliferation, faster wound closure, and improved outcomes compared to conventional treatments [18,19,69]. Taken together, these results suggest that *Psidium guajava* leaf extract-loaded nanofibers offer a promising therapeutic approach for effective wound management.

#### 3.6.2. Histopathology

**Figure 7:**
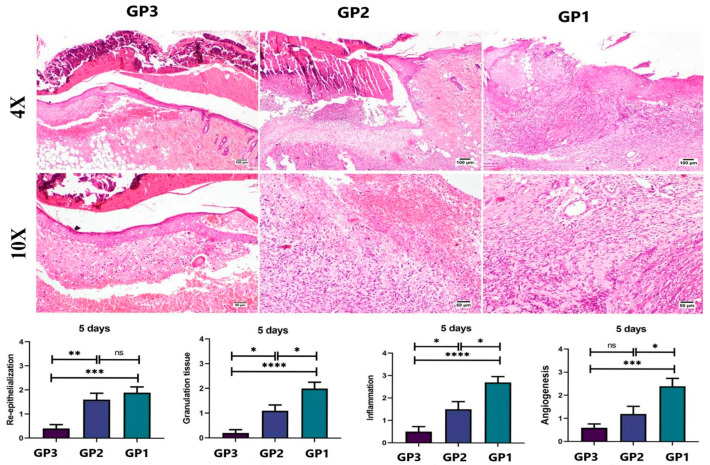
Histopathological examination of tissue samples from the skin wound collected on day 5 from the different groups (H&E). Charts show histological scores across groups on day 5 (*n* = 3 per group). Data expressed as means ± SEM, ns: non-significant difference, * *p* < 0.05, ** *p* < 0.01, *** *p* < 0.001, and **** *p* < 0.0001.

On the fifth day (Figure 7), a microscopic examination of skin samples from the control group (GP3) revealed a thick crust covering the wound area, with partial retarded epithelization, an intense inflammatory reaction, and extensive hemorrhage. The Panthenol cream-treated group (GP2) showed signs similar to those in the control group, with only mild improvement, as evidenced by early re-epithelization, haphazardly arranged granulation tissue, and reduced inflammation. Regarding the nanofiber-treated group (GP1), signs of healing were evident, with minimal exudation and hemorrhage. Re-epithelization was observed at the wound edges, angiogenesis was evident, and inflammation had subsided to some extent. The healing score on the 5th day showed significant improvement in both treated groups compared with the control group (GP3). The best healing score was noticed in the group treated with the nanofiber (GP1).

**Figure 8:**
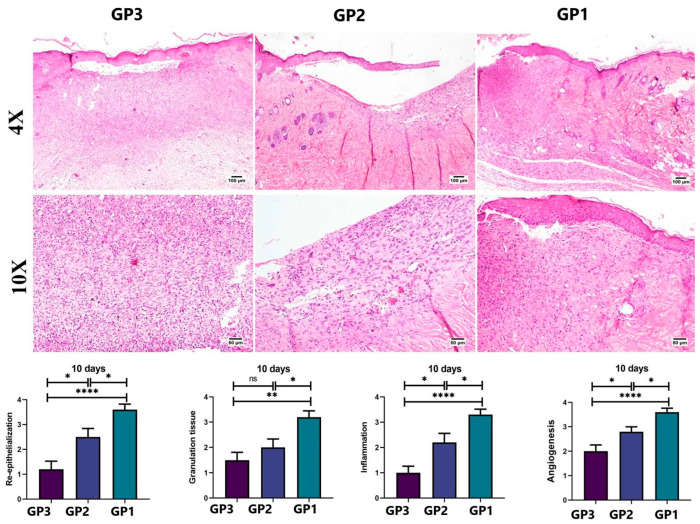
Histopathological examination of tissue samples from the skin wound collected on day 10 from the different groups (H&E). Charts show histological scores across groups on day 10 (*n* = 3 per group). Data expressed as means ± SEM, ns: non-significant difference, * *p* < 0.05, ** *p* < 0.01, and **** *p* < 0.0001.

**Figure 9:**
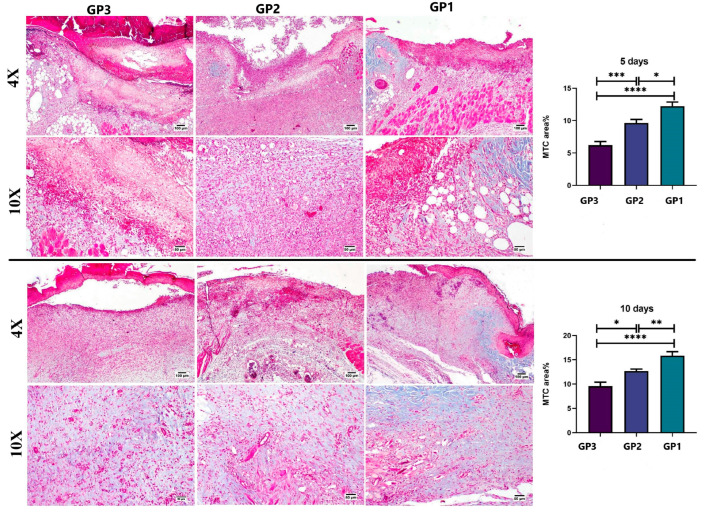
Masson’s trichrome staining of skin sections derived from different groups on days 5 and 14 post-injury. Charts show the area % of bluish-stained sections in different groups on days 5 and 10 (*n* = 3 per group). Data expressed as means ± SEM, * *p* < 0.05, ** *p* < 0.01, *** *p* < 0.001, and **** *p* < 0.0001.

On the 10th day (Figure 8), the wound gap in all groups was filled by granulation tissue, and the upper surface was covered entirely by newly formed epithelium. The control group (GP3) showed a thin epithelial cover, a granulation tissue core, and an inflammatory reaction. The Panthenol cream-treated group exhibited organized tissue, filling the wound gap with less inflammatory reaction. The nanofiber-treated group (GP1) showed healthy, noninflamed, organized tissue, with more collagen filling the wound area and a complete epithelial cover. The healing score was significantly lower in the control group (GP3) than in both treated groups. The nanofiber-treated group had a higher healing score than the Panthenol cream group across all estimated parameters. Regarding collagen content (Figure 9), both treated groups showed a significant increase compared to the control group at both time points. The nanofiber-treated group showed a significant increase in collagen formation compared with the Panthenol cream group (GP2).

These outcomes confirmed that *Psidium guajava* leaf extract exhibits notable wound-healing effects, primarily due to its high content of phenolic compounds and other bioactives. Although in the current study the *Psidium guajava* leaf extract was characterized only by its total phenolic and flavonoid contents, its composition is expected to be consistent with reported phytochemical analyses of *Psidium guajava* leaves, which report high levels of phenolic acids (such as gallic, chlorogenic, and caffeic acids), flavan-3-ols (catechin, epicatechin), and flavonol glycosides including quercetin, rutin, avicularin, isoquercitrin, and kaempferol derivatives [70,71]. These components confer potent antioxidant and anti-inflammatory properties, thereby enhancing tissue regeneration and protecting the wound bed against oxidative damage [19]. Thus, the total polyphenol and flavonoid values reported in the current study reflect the combined contribution of these bioactive constituents to the observed enhancement in wound closure and tissue regeneration. Topical application of gallic acid, for instance, has demonstrated significant wound healing activity through its antioxidant and anti-inflammatory actions in animal models. Flavonoids present in *Psidium guajava* promote collagen synthesis and stabilization, facilitating the crosslinking of collagen fibers, reducing the degradation of soluble collagen, and accelerating its conversion to insoluble forms, all of which are vital for effective wound repair. Anthocyanins, a class of flavonoids found in *Psidium guajava* leaves, are recognized for their antioxidant and anti-inflammatory activities, including inhibition of cyclooxygenase and promotion of tissue repair, contributing further to wound healing [72,73].

Moreover, our histopathological findings demonstrated the role of the used nanofiber in wound healing, as it helped stop bleeding, promoted collagen fiber formation, and improved re-epithelization, leading to complete wound closure. Collagen fibers are the most important extracellular matrix component in wound healing, so the increased collagen deposition observed in nanofiber-treated wounds confirms their ability to improve the healing process.

#### 3.6.3. Detection of Cytokines and Growth Factors

MMPs are critical regulators present in both acute and chronic wounds, playing a pivotal role in the degradation and deposition of the extracellular matrix required for wound reepithelialization. Properly timed activation of MMPs in response to tissue injury is essential for successful healing, as these enzymes participate in the removal of damaged matrix components, cell migration, and angiogenesis throughout the wound-healing cascade. MMPs are classified into several families based on substrate specificity and share extensive homology within each family. While controlled MMP activity promotes healing, excessive protease activity can lead to impaired wound closure and chronic nonhealing wounds [74,75]. In the present study, treatment with the *Psidium guajava* leaf extract-loaded nanofiber resulted in a significant reduction in protease activity at day 10 post-wounding compared with the Panthenol cream and distilled water control groups. This modulation of MMPs is consistent with improved matrix remodeling and accelerated tissue repair.

**Figure 10:**
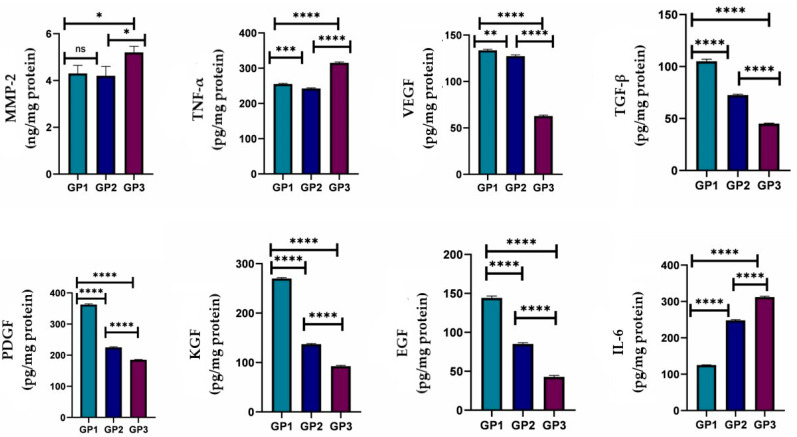
Cytokines and growth factors from different groups on day 5, ns; non-significant difference (*n* = 6 per group),* *p* < 0.05, ** *p* < 0.01, *** *p* < 0.001, and **** *p* < 0.0001.

**Figure 11:**
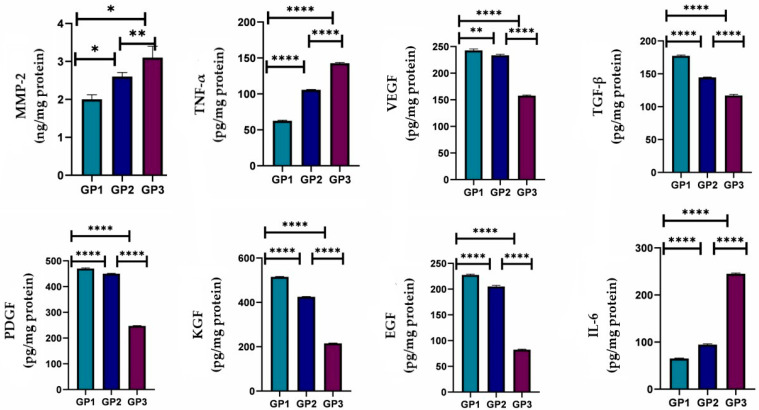
Cytokines and growth factors from different groups on day 10 (*n* = 6 per group), * *p* < 0.05, ** *p* < 0.01, and **** *p* < 0.0001.

Growth factors are essential mediators of tissue regeneration, displaying chemotactic, mitogenic, angiogenic, and matrix-modulating effects. They play diverse roles in wound healing by attracting cells to the injury site, promoting cellular proliferation, and regulating extracellular matrix synthesis and breakdown [76]. The expression of specific growth factors, such as PDGF, VEGF, TGF-β, KGF, and EGF, was significantly increased in wounds treated with the *Psidium guajava* leaf extract-loaded nanofiber, which correlated with enhanced wound repair compared to the reference treatments, as illustrated in Figure 10 and Figure 11. PDGF, one of the earliest-discovered growth factors, is produced by platelets, macrophages, and other cell types and acts as a potent mitogen and chemotactic agent for fibroblasts, smooth muscle cells, and other essential cells involved in wound healing. VEGF, which shares homology with PDGF, is a potent stimulatory factor for angiogenesis. TGF-β is known for its ability to drive collagen deposition and modulate inflammation, while KGF and EGF are critical for epithelial regeneration.

Additionally, the study observed that treatment with Panthenol cream was associated with elevated pro-inflammatory cytokines, notably TNF-α and IL-6, at day 10. In contrast, the *Psidium guajava* leaf extract-loaded nanofiber resulted in lower cytokine levels, consistent with attenuated inflammation and improved healing. TNF-α, secreted by macrophages, helps induce extracellular matrix protein synthesis, whereas excessive and prolonged cytokine release can contribute to chronic inflammation and delayed wound closure. Altogether, the present findings highlight the beneficial effects of the *Psidium guajava* leaf extract-loaded nanofiber in modulating protease activity and growth factor expression, resulting in substantial improvements in wound-healing outcomes compared with standard treatments.

The superior healing attained with the *Psidium guajava* leaf extract-loaded nanofiber compared to commercial cream (Panthenol) suggests a beneficial effect of the bioactive extract in a supportive nanofibrous matrix; however, future studies should include a blank nanofiber-treated group to assess the independent contribution of the nanofiber and to confirm the specific added value of the loaded extract. Therefore, the limitation of the current study is the absence of a control group treated with a blank nanofiber to evaluate the relative contribution of the *Psidium guajava* leaf extract versus the nanofibrous scaffold itself to the observed acceleration of wound closure. Furthermore, the current study primarily investigated the efficacy of the *Psidium guajava* leaf extract-loaded nanofiber dressing in promoting wound healing, and no safety studies were performed. Despite the absence of apparent local adverse effects, such as erythema, edema, or necrosis, and no systemic signs of distress were noticed in the treated animals. However, comprehensive biocompatibility studies will be required in future studies before this nanofiber formulation can be considered for clinical application in humans.

## 4. Conclusions

The current study demonstrates that the extraction method can affect the yield and concentration of bioactive compounds within herbal extracts. It has also been observed that extraction temperature and solvent type influence total polyphenol and flavonoid content; thus, decoction of *Psidium guajava* leaves with distilled water at high temperatures could increase the yield of polyphenols and flavonoids. Moreover, our results showed that the freeze-drying process significantly impacted the yield of extracted polyphenols and flavonoids, without altering their chemical structure. Additionally, lower degradation and higher polyphenol concentrations were observed. Moreover, the current study demonstrated the development of a novel therapeutic strategy for wound treatment through two pathways: the first is to control inflammation by decreasing levels of inflammatory cytokines (TNF-α and IL-6 in tissues), and the second is to increase levels of tissue growth factors, which promote wound healing. Finally, the commercial potential of botanical electrospun nanofibers requires advancements in fundamental research and the establishment of technologies for large-scale manufacturing.

## Figures and Tables

**Table 1:** In vivo wound healing assay utilizing botanical nanofiber and commercial cream.

Group Name	Treatment
GP 1	Nanofibers loaded with *Psidium guajava* leaf extract
GP2	Panthenol cream
GP 3	Distilled water

**Table 2:** Experimental runs of the 2^3^ factorial design.

RUN	A: Vacuum X_1_ (bar)	B: Freezing Time at −18 °C X_2_ (h)	C: Lyophilization Time X_3_ (h)	Polyphenols Y_1_ (mg GAE/g)	Flavonoids Y_2_ (mg RE/g)
1	0.01	24.00	48.00	476.90 ± 3.8	171.5 ± 3.6
2	0.01	36.00	36.00	462.16 ± 3.4	149.02 ± 1.5
3	0.01	24.00	24.00	490.00 ± 3.8	125.00 ± 1.9
4	0.02	48.00	24.00	451.00 ± 0.2	166.13 ± 2.5
5	0.02	24.00	24.00	448.34 ± 2.1	123.33 ± 2.5
6	0.02	24.00	48.00	506.70 ± 0.7	152.25 ± 1.7
7	0.02	24.00	24.00	451.33 ± 0.5	125.81 ± 1.3
8	0.02	24.00	48.00	520.75 ± 2.8	169.54 ± 3.6
9	0.01	48.00	48.00	439.66 ± 0.4	164.25 ± 2.3
10	0.02	48.00	48.00	477.08 ± 1.7	208.25 ± 1.5
11	0.01	24.00	24.00	479.47 ± 1.5	133.00 ± 1.4
12	0.01	24.00	48.00	480.38 ± 2.1	177.36 ± 2.6
13	0.02	48.00	48.00	467.16 ± 3.5	176.22 ± 3.0
14	0.01	36.00	36.00	474.06 ± 3.5	143.12 ± 1.8
15	0.01	48.00	48.00	478.13 ± 1.4	160.42 ± 3.2
16	0.02	48.00	24.00	458.28 ± 4.9	169.04 ± 2.1
17	0.01	48.00	24.00	408.87 ± 0.7	119.75 ± 1.1
18	0.01	48.00	24.00	405.92 ± 2.2	122.59 ± 2.5

**Table 3:** Output of the 2^3^ factorial design.

Responses	Polyphenol Content (mg GAE/g)	Flavonoid Content (mg RE/g)
Adjusted *R*^2^	0.873	0.870
Predicted *R*^2^	0.727	0.708
Adequate precision	15.39	11.02

**Table 4:** Surface roughness measurement containing roughness average [R_a_], root mean square roughness [R_q_], the maximum height of the roughness [R_t_], maximum roughness peak height [R_p_], and an average maximum height of the roughness [R_tm_].

Samples	R_a_ (nm)	R_q_ (nm)	R_t_ (nm)	R_p_ (nm)	R_tm_ (nm)
Unloaded nanofiber	49.37	63.42	522.49	300.44	406.39
Loaded nanofiber	45.36	59.26	496.47	189.85	393.57

## Data Availability

Data is contained within the article.

## References

[1] Wan Z., Liu Q., Zhe Y., Li J., Ding D., Liu S., Wang H., Qiao H., Yang J., Zhang S. (2025). Single-atom nanozymes with intelligent response to pathological microenvironments for bacterially infected wound healing. Biomater. Sci..

[2] El Ghacham S., Hejji L., Aoulad El Hadj Ali Y., Wahby A., Tamegart L., Pérez-Villarejo L., Mennane Z., Souhail B., Azzouz A. (2025). Enhanced antibacterial and wound healing efficacy of a novel CQDs@AgNPs@CS-based nanocomposites: A multifunctional approach for advanced wound care. Int. J. Biol. Macromol..

[3] Sen C.K. (2021). Human wound and its burden: Updated 2020 compendium of estimates. Adv. Wound Care.

[4] Souliotis K., Kalemikerakis I., Saridi M., Papageorgiou M., Kalokerinou A. (2016). A cost and clinical effectiveness analysis among moist wound healing dressings versus traditional methods in home care patients with pressure ulcers. Wound Repair Regen..

[5] Lagoa T., Queiroga M.C., Martins L. (2024). An Overview of Wound Dressing Materials. Pharmaceuticals.

[6] Zhou R., Ma Y., Yang M., Cheng Y., Ma X., Li B., Zhang Y., Cui X., Liu M., Long Y. (2025). Wound dressings using electrospun nanofibers: Mechanisms, applications, and future directions. Eur. Polym. J..

[7] Almukainzi M., El-Masry T.A., Negm W.A., Elekhnawy E., Saleh A., Sayed A.E., Ahmed H.M., Abdelkader D.H. (2022). Co-delivery of gentiopicroside and thymoquinone using electrospun m-PEG/PVP nanofibers: In-vitro and In vivo studies for antibacterial wound dressing in diabetic rats. Int. J. Pharm..

[8] Akombaetwa N., Bwanga A., Makoni P.A., Witika B.A. (2022). Applications of Electrospun Drug-Eluting Nanofibers in Wound Healing: Current and Future Perspectives. Polymers.

[9] Balogh-Weiser D., Molnár A., Tóth G.D., Koplányi G., Szemes J., Decsi B., Katona G., Salamah M., Ender F., Kovács A. (2023). Combined Nanofibrous Face Mask: Co-Formulation of Lipases and Antibiotic Agent by Electrospinning Technique. Pharmaceutics.

[10] Blaj D.A., Peptu C.A., Danu M., Harabagiu V., Peptu C., Bujor A., Ochiuz L., Tuchiluș C.G. (2024). Enrofloxacin Pharmaceutical Formulations through the Polymer-Free Electrospinning of β-Cyclodextrin-oligolactide Derivatives. Pharmaceutics.

[11] Khan A.u.R., Morsi Y., Zhu T., Ahmad A., Xie X., Yu F., Mo X. (2021). Electrospinning: An emerging technology to construct polymer-based nanofibrous scaffolds for diabetic wound healing. Front. Mater. Sci..

[12] Mani M.P., Ponnambalath Mohanadas H., Mohd Faudzi A.A., Ismail A.F., Tucker N., Mohamaddan S., Ayyar M., Palanisamy T., Rathanasamy R., Jaganathan S.K. (2024). Characterization and Performance Evaluation of Magnesium Chloride-Enriched Polyurethane Nanofiber Patches for Wound Dressings. Int. J. Nanomed..

[13] Jaganathan S.K., Mani M.P. (2018). Electrospun polyurethane nanofibrous composite impregnated with metallic copper for wound-healing application. 3 Biotech.

[14] Mitra A., Shahid A., Kumari S., Mukherjee T., Pramanick S., Mohanty S., Ansari M.A., Adhikary K., Prabhakar P.K., Kesari K.K. (2025). Optimizing wound healing: Insights from phytochemicals and advanced therapies. Inflammopharmacology.

[15] Kmail A., Said O., Saad B. (2023). How Thymoquinone from Nigella sativa Accelerates Wound Healing through Multiple Mechanisms and Targets. Curr. Issues Mol. Biol..

[16] Abdellatif M.M., Elakkad Y.E., Elwakeel A.A., Allam R.M., Mousa M.R. (2021). Formulation and characterization of propolis and tea tree oil nanoemulsion loaded with clindamycin hydrochloride for wound healing: In-vitro and in-vivo wound healing assessment. Saudi Pharm. J..

[17] Yang F., Fang Z., Yang M., Cheng Z., Tian Y., Liang J., Li T. (2025). Research Hotspots in Natural Products for Wound Healing: A Bibliometric Analysis and Literature Review. Clin. Cosmet. Investig. Dermatol..

[18] Bilal K., Mehboob F., Akhtar N., Mirza I.A., Okla M.K., Dar M.J., Saleh I.A., Zomot N., Fatima H. (2024). Wound healing, antioxidant and antibacterial activities of polyphenols of *Psidium guajava* L. leaves. S. Afr. J. Bot..

[19] Ghaderi F., Ebrahimi E., Sari Aslani F., Koohi-Hosseinabadi O., Koohpeyma F., Irajie C., Tanideh N., Iraji A. (2022). The effect of hydroalcoholic extract of *Psidium guajava* L. on experimentally induced oral mucosal wound in rat. BMC Complement. Med. Ther..

[20] Salunke M.R., Shinde V. (2025). Molecular insights and efficacy of guava leaf oil emulgel in managing non diabetic as well as diabetic wound healing by reducing inflammation and oxidative stress. Inflammopharmacology.

[21] Kumar M., Tomar M., Amarowicz R., Saurabh V., Nair M.S., Maheshwari C., Sasi M., Prajapati U., Hasan M., Singh S. (2021). Guava (*Psidium guajava* L.) Leaves: Nutritional Composition, Phytochemical Profile, and Health-Promoting Bioactivities. Foods.

[22] Gutierrez Montiel D., Guerrero Barrera A.L., Martínez Ávila G.C.G., Gonzalez Hernandez M.D., Chavez Vela N.A., Avelar Gonzalez F.J., Ramírez Castillo F.Y. (2023). Influence of the Extraction Method on the Polyphenolic Profile and the Antioxidant Activity of *Psidium guajava* L. Leaf Extracts. Molecules.

[23] Seo J., Lee S., Elam M.L., Johnson S.A., Kang J., Arjmandi B.H. (2014). Study to find the best extraction solvent for use with guava leaves (*Psidium guajava* L.) for high antioxidant efficacy. Food Sci. Nutr..

[24] Thi N.-D.D., Nguyen M.-T., Bui Thi B.-H., Mai Q.-Q., Nguyen P.-V., Nguyen Q.-V. (2024). Optimizing Polyphenolic Extraction from Wild Guava Leaves: A Response Surface Methodology Approach to Antioxidant and α-Glucosidase Inhibitory Activities. Nat. Prod. Commun..

[25] Fang Z., Bhandari B. (2010). Encapsulation of polyphenols—A review. Trends Food Sci. Technol..

[26] ElNaker N.A., Daou M., Ochsenkühn M.A., Amin S.A., Yousef A.F., Yousef L.F. (2021). A metabolomics approach to evaluate the effect of lyophilization versus oven drying on the chemical composition of plant extracts. Sci. Rep..

[27] Jovanović A.A., Lević S.M., Pavlović V.B., Marković S.B., Pjanović R.V., Đorđević V.B., Nedović V., Bugarski B.M. (2021). Freeze vs. Spray Drying for Dry Wild Thyme (*Thymus serpyllum* L.) Extract Formulations: The Impact of Gelatin as a Coating Material. Molecules.

[28] Burman V., Kanaujia H., Lehari K., Naresh Singh P. (2019). Characterization of phenolic compounds of turmeric using TLC. J. Pharmacogn. Phytochem..

[29] Attard E. (2013). A rapid microtitre plate Folin-Ciocalteu method for the assessment of polyphenols. Cent. Eur. J. Biol..

[30] Herald T.J., Gadgil P., Tilley M. (2012). High-throughput micro plate assays for screening flavonoid content and DPPH-scavenging activity in sorghum bran and flour. J. Sci. Food Agric..

[31] Simao A.A., Marques T.R., Marcussi S., Correa A.D. (2017). Aqueous extract of *Psidium guajava* leaves: Phenolic compounds and inhibitory potential on digestive enzymes. An. Acad. Bras. Cienc..

[32] de Araújo A.A., Soares L.A.L., Assunção Ferreira M.R., de Souza Neto M.A., da Silva G.R., de Araújo R.F., Guerra G.C.B., de Melo M.C.N. (2014). Quantification of polyphenols and evaluation of antimicrobial, analgesic and anti-inflammatory activities of aqueous and acetone–water extracts of Libidibia ferrea, Parapiptadenia rigida and *Psidium guajava*. J. Ethnopharmacol..

[33] Albash R., Ali S.K., Abdelmonem R., Agiba A.M., Aldhahri R., Saleh A., Kassem A.B., Abdellatif M.M. (2025). Electrospun Nanofiber-Scaffold-Loaded Levocetirizine Dihydrochloride Cerosomes for Combined Management of Atopic Dermatitis and Methicillin-Resistant Staphylococcus Aureus (MRSA) Skin Infection: In Vitro and In Vivo Studies. Pharmaceuticals.

[34] Teaima M.H., Abdelnaby F.A., Fadel M., El-Nabarawi M.A., Shoueir K.R. (2020). Synthesis of Biocompatible and Environmentally Nanofibrous Mats Loaded with Moxifloxacin as a Model Drug for Biomedical Applications. Pharmaceutics.

[35] Abdelmonem R., Bakr A., Badawy I., Abd El Maksoud A.I., Attia R.T. (2024). Quality by Design Approach for the Formulation and Evaluation of Stem Cells Derived Rosmarinic Acid-Loaded Nanofibers as an Anti-Wrinkle Patch: In Vitro and In Vivo Characterizations. Pharmaceutics.

[36] Pinto M.L., Pires J., Carvalho A.P., de Carvalho M.B., Bordado J.C. (2006). Synthesis and regeneration of polyurethane/adsorbent composites and their characterization by adsorption methods. Microporous Mesoporous Mater..

[37] Bancroft J.D., Gamble M. (2008). Theory and Practice of Histological Techniques.

[38] Bakr R.O., Amer R.I., Attia D., Abdelhafez M.M., Al-Mokaddem A.K., El-Gendy A.E.-N.G., El-Fishawy A.M., Fayed M.A.A., Gad S.S. (2021). In-vivo wound healing activity of a novel composite sponge loaded with mucilage and lipoidal matter of Hibiscus species. Biomed. Pharmacother..

[39] Bhagya Raj G.V.S., Dash K.K. (2020). Ultrasound-assisted extraction of phytocompounds from dragon fruit peel: Optimization, kinetics and thermodynamic studies. Ultrason. Sonochem..

[40] Zhang Q.-W., Lin L.-G., Ye W.-C. (2018). Techniques for extraction and isolation of natural products: A comprehensive review. Chin. Med..

[41] Lima A.M., Siani A.C., Nakamura M.J., D’Avila L.A. (2015). Selective and cost-effective protocol to separate bioactive triterpene acids from plant matrices using alkalinized ethanol: Application to leaves of Myrtaceae species. Pharmacogn. Mag..

[42] de Novais N.S., de Souza Ribeiro M.M., Viganó J., Coelho D.B., Falcão L.d.S., de Moraes M.A., Veggi P.C. (2023). High- and low-pressure fixed bed extraction behaviors to obtain phenolic compounds from barbatimão (*Stryphnodendron adstringens*) bark. Sustain. Chem. Pharm..

[43] Galanakis C.M., Galanakis C.M. (2015). Chapter 3—The universal recovery strategy. Food Waste Recovery.

[44] Złotek U., Mikulska S., Nagajek M., Świeca M. (2016). The effect of different solvents and number of extraction steps on the polyphenol content and antioxidant capacity of basil leaves (*Ocimum basilicum* L.) extracts. Saudi J. Biol. Sci..

[45] Nayak A., Bhushan B., Rosales A., Turienzo L.R., Cortina J.L. (2018). Valorisation potential of Cabernet grape pomace for the recovery of polyphenols: Process intensification, optimisation and study of kinetics. Food Bioprod. Process..

[46] Chu J., Ming Y., Cui Q., Zheng N., Yang S., Li W., Gao H., Zhang R., Cheng X. (2022). Efficient extraction and antioxidant activity of polyphenols from Antrodia cinnamomea. BMC Biotechnol..

[47] Wang L., Wu Y., Liu Y., Wu Z. (2017). Complex Enzyme-Assisted Extraction Releases Antioxidative Phenolic Compositions from Guava Leaves. Molecules.

[48] Shang Y.-F., Hao W.-D., Zhang W., Ma Y.-L., Niu X.-L., Wei Z.-J., Sun S.-Q., Xu J.-L. (2024). Non-targeted LC-MS Metabolite profiling: Contrasting water hydrodistillation and methanol extraction of *Rose damascens* Mill. LWT.

[49] Baron G., Ferrario G., Marinello C., Carini M., Morazzoni P., Aldini G. (2021). Effect of Extraction Solvent and Temperature on Polyphenol Profiles, Antioxidant and Anti-Inflammatory Effects of Red Grape Skin By-Product. Molecules.

[50] Lorena C., Ressaissi A., Serralheiro M.L. (2022). Bioactives from *Psidium guajava* leaf decoction: LC-HRMS-MS-Qtof identification, bioactivities and bioavailability evaluation. Food Chem. Adv..

[51] Wang L., Wen H., Yang N., Li H. (2023). Effect of vacuum freeze drying and hot air drying on dried mulberry fruit quality. PLoS ONE.

[52] Chen H., Cheng Y.-x., Cheng W.-l. (2025). Simplified model for heat and mass transfer during primary drying of dual-chamber cartridge with shell holder. Int. J. Heat Mass Transf..

[53] Nowicka P., Lech K., Wojdyło A. (2025). The influence of different drying techniques on polyphenols profile (LC–MS-PDA-Q/TOF) of peach fruit and their pro-health properties by in vitro. Sci. Rep..

[54] Feng S., Bi J., Laaksonen T., Laurén P., Yi J. (2024). Texture of freeze-dried intact and restructured fruits: Formation mechanisms and control technologies. Trends Food Sci. Technol..

[55] Pérez-Gregorio M.R., Regueiro J., González-Barreiro C., Rial-Otero R., Simal-Gándara J. (2011). Changes in antioxidant flavonoids during freeze-drying of red onions and subsequent storage. Food Control.

[56] Demir E., Tappi S., Dymek K., Rocculi P., Gómez Galindo F. (2023). Reversible electroporation caused by pulsed electric field—Opportunities and challenges for the food sector. Trends Food Sci. Technol..

[57] Tan S., Tang J., Shi W., Wang Z., Xiang Y., Deng T., Gao X., Li W., Shi S. (2020). Effects of three drying methods on polyphenol composition and antioxidant activities of Litchi chinensis Sonn. Food Sci. Biotechnol..

[58] Babaei Rad S., Mumivand H., Mollaei S., Khadivi A. (2025). Effect of drying methods on phenolic compounds and antioxidant activity of *Capparis spinosa* L. fruits. BMC Plant Biol..

[59] Butt E., Altemimi A.B., Younas A., Butt M.S., Jalal M., Bhatty M., Abdi G., Aadil R.M. (2025). Guava (*Psidium guajava*): A brief overview of its therapeutic and health potential. Food Chem. X.

[60] Guo S., Wang P., Song P., Li N. (2022). Electrospinning of botanicals for skin wound healing. Front. Bioeng. Biotechnol..

[61] Garcia-Orue I., Gainza G., Garcia-Garcia P., Gutierrez F.B., Aguirre J.J., Hernandez R.M., Delgado A., Igartua M. (2019). Composite nanofibrous membranes of PLGA/Aloe vera containing lipid nanoparticles for wound dressing applications. Int. J. Pharm..

[62] Homaeigohar S., Tsai T.-Y., Zarie E.S., Elbahri M., Young T.-H., Boccaccini A.R. (2020). Bovine Serum Albumin (BSA)/polyacrylonitrile (PAN) biohybrid nanofibers coated with a biomineralized calcium deficient hydroxyapatite (HA) shell for wound dressing. Mater. Sci. Eng. C.

[63] Ai C., Liu S., Zhao Y., Huang X., Teng H., Li S., Chen L. (2024). Hybrid electrospinning of pullulan and citrus pectin to load astaxanthin: Preparation, characterization, and functional evaluation. Food Chem. X.

[64] Sasan S., Molavi A.M., Moqadam K.H., Farrokhi N., Oroojalian F. (2024). Enhanced wound healing properties of biodegradable PCL/alginate core-shell nanofibers containing Salvia abrotanoides essential oil and ZnO nanoparticles. Int. J. Biol. Macromol..

[65] Alizadeh M., Salehi S., Tavakoli M., Mirhaj M., Varshosaz J., Kazemi N., Salehi S., Mehrjoo M., Abadi S.A.M. (2023). PDGF and VEGF-releasing bi-layer wound dressing made of sodium tripolyphosphate crosslinked gelatin-sponge layer and a carrageenan nanofiber layer. Int. J. Biol. Macromol..

[66] Du P., Chen X., Chen Y., Li J., Lu Y., Li X., Hu K., Chen J., Lv G. (2023). In vivo and in vitro studies of a propolis-enriched silk fibroin-gelatin composite nanofiber wound dressing. Heliyon.

[67] Parthasarathy V., Kumar P.S., Aureen Albert A., Krishnasamy S., Chandrasekar M. (2025). Recent progress in nanocellulose-based biocomposites for bone tissue engineering and wound healing applications. Carbohydr. Polym..

[68] Fernandes K.P.S., Bussadori S.K., Marques M.M., Wadt N.S.Y., Bach E., Martins M.D. (2010). Healing and cytotoxic effects of *Psidium guajava* (Myrtaceae) leaf extracts. Braz. J. Oral Sci..

[69] Chah K.F., Eze C.A., Emuelosi C.E., Esimone C.O. (2006). Antibacterial and wound healing properties of methanolic extracts of some Nigerian medicinal plants. J. Ethnopharmacol..

[70] Kamble M.T., Chaiyapechara S., Salin K.R., Bunphimpapha P., Chavan B.R., Bhujel R.C., Medhe S.V., Kettawan A., Thiyajai P., Thompson K.D. (2024). Guava and Star gooseberry leaf extracts improve growth performance, innate immunity, intestinal microbial community, and disease resistance in Nile tilapia (*Oreochromis niloticus*) against Aeromonas hydrophila. Aquac. Rep..

[71] Hsieh C.-L., Yang M.-H., Chyau C.-C., Chiu C.-H., Wang H.-E., Lin Y.-C., Chiu W.-T., Peng R.Y. (2007). Kinetic analysis on the sensitivity of glucose- or glyoxal-induced LDL glycation to the inhibitory effect of *Psidium guajava* extract in a physiomimic system. Biosystems.

[72] Kim M.-H., Kim J.N., Han S.N., Kim H.-K. (2015). Ursolic acid isolated from guava leaves inhibits inflammatory mediators and reactive oxygen species in LPS-stimulated macrophages. Immunopharmacol. Immunotoxicol..

[73] Tanideh N., Rostami Nasab M.A., Hasssanpour I., Koohi Hosseinabadi O., Mojahed M., Nabavizadeh S.S. (2020). valuation of *Psidium guajava* L. Leaf Oil Extract Effect on Induced Osteoarthritis in Male Rats. Iran. J. Orthop. Surg..

[74] Sabino F., auf dem Keller U. (2015). Matrix metalloproteinases in impaired wound healing. Met. Med..

[75] Stevens L.J., Page-McCaw A. (2012). A secreted MMP is required for reepithelialization during wound healing. Mol. Biol. Cell.

[76] Ayuk S.M., Abrahamse H., Houreld N.N. (2016). The Role of Matrix Metalloproteinases in Diabetic Wound Healing in relation to Photobiomodulation. J. Diabetes Res..

